# Improving astaxanthin production in *Escherichia coli* by co-utilizing CrtZ enzymes with different substrate preference

**DOI:** 10.1186/s12934-022-01798-1

**Published:** 2022-04-25

**Authors:** Meng Zhang, Zhongkuo Gong, Jinlei Tang, Fuping Lu, QingYan Li, XueLi Zhang

**Affiliations:** 1 College of Biotechnology, Tianjin University of Sciences and Technology, Tianjin, 300457 China; 2 Tianjin Institute of Industrial Biotechnology, Chinese of Academy of Sciences, 32 Xiqidao, Tianjin Airport Economic Park, Tianjin, 300308 China; 3 Key Laboratory of Systems Microbial Biotechnology, Chinese Academy of Sciences, Tianjin, 300308 China; 4 National Technology Innovation Center of Synthetic Biology, Tianjin, 300308 China; 5 University of Chinese Academy of Sciences, Beijing, 100071 China

**Keywords:** *Escherichia coli*, Astaxanthin, β-carotene hydroxylase, Substrate preference, Combined utilization

## Abstract

**Background:**

The bifunctional enzyme β-carotene hydroxylase (CrtZ) catalyzes the hydroxylation of carotenoid β-ionone rings at the 3, 3’ position regardless of the presence of keto group at 4, 4’ position, which is an important step in the synthesis of astaxanthin. The level and substrate preference of CrtZ may have great effect on the amount of astaxanthin and the accumulation of intermediates.

**Results:**

In this study, the substrate preference of PC*crtZ* from *Paracoccus* sp. PC1 and PA*crtZ* from *Pantoea Agglomerans* were certified and were combined utilization for increase astaxanthin production. Firstly, PC*crtZ* from *Paracoccus* sp. PC1 and PA*crtZ* from *P. Agglomerans* were expressed in platform strains CAR032 (β-carotene producing strain) and Can004 (canthaxanthin producing strain) separately to identify their substrate preference for carotenoids with keto groups at 4,4’ position or not. The results showed that PC*crtZ* led to a lower zeaxanthin yield in CAR032 compared to that of PA*crtZ*. On the contrary, higher astaxanthin production was obtained in Can004 by *PCcrtZ* than that of PA*crtZ*. This demonstrated that PCCrtZ has higher canthaxanthin to astaxanthin conversion ability than PACrtZ, while PACrtZ prefer using β-carotene as substrate. Finally, Ast010, which has two copies of PA*crtZ* and one copy of PC*crtZ* produced 1.82 g/L of astaxanthin after 70 h of fed-batch fermentation.

**Conclusions:**

Combined utilization of *crtZ* genes, which have β-carotene and canthaxanthin substrate preference respectively, can greatly enhance the production of astaxanthin and increase the ratio of astaxanthin among total carotenoids.

**Supplementary information:**

The online version contains supplementary material available at 10.1186/s12934-022-01798-1.

## Background

Carotenoids are organic pigments ranging in color from yellow to red that are naturally produced by certain organisms, including photosynthetic organisms (e.g. plants, algae, cyanobacteria), as well as some nonphotosynthetic bacteria, yeast and fungi. Carotenoids such as lutein, zeaxanthin or astaxanthin are important additives in food and feed industry as pigmenting substances and precursors of vitamin A derivatives [1–4]. In addition, carotenoids have health-promoting effects such as enhancing the immune response and, due to their antioxidant properties, anticancer activity, which makes them of interest as nutraceuticals [1, 5]. An economical process for preparing carotenoids and foodstuffs with an increased carotenoid content is therefore of great importance. Producing carotenoids by metabolic engineering of recombinant microorganisms gained increasing attention because it is environmental friendly and has a lower cost than chemical synthesis.

**Fig. 1:**
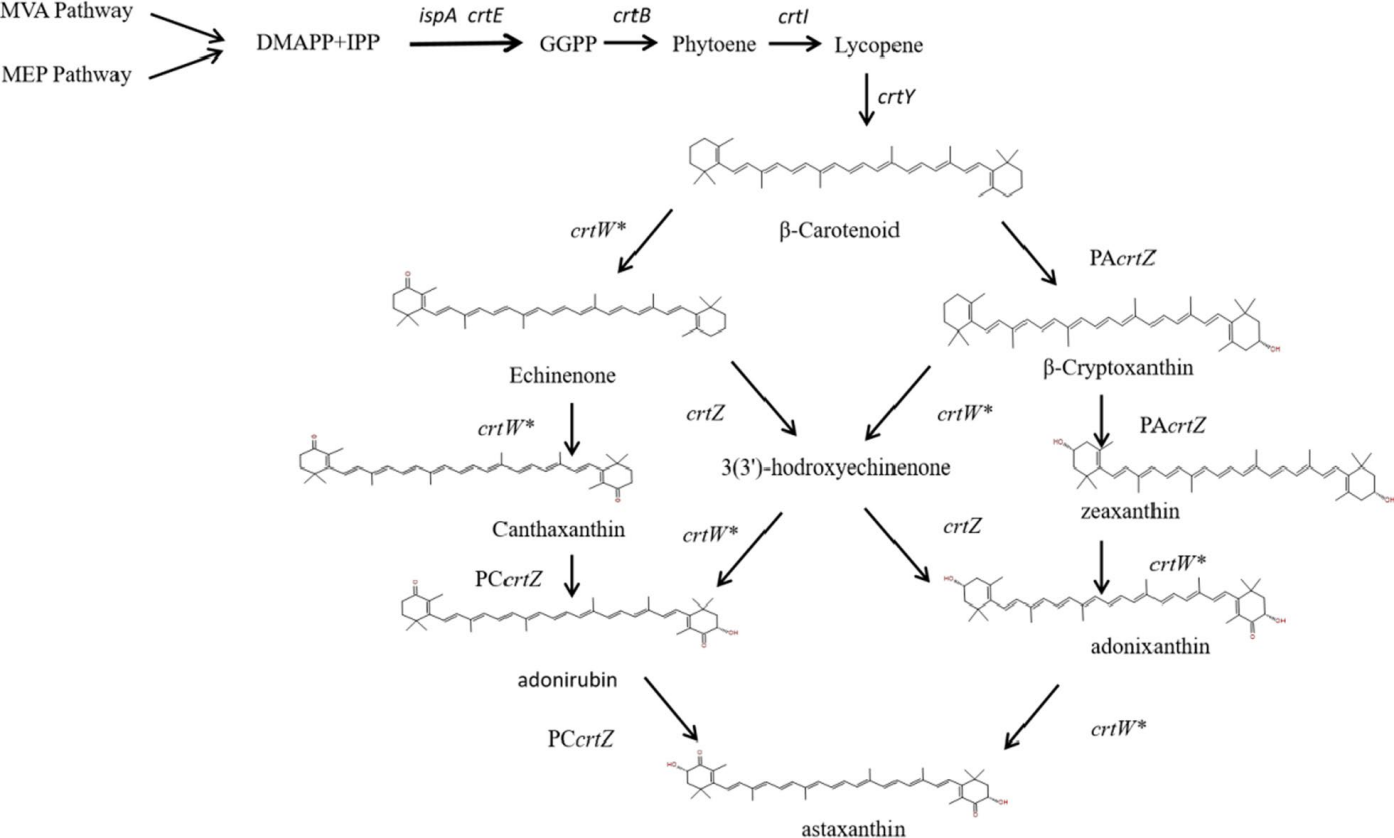
Schematic diagram of the heterologous synthesis pathway for the production of carotenoids. *MVA Pathway* mevalonic acid pathway, *MEP Pathway* 2-methyl-D-erythritol-4-phosphate pathway, *DMAPP* dimethylallyl pyrophosphate, *IPP* isopentenyl pyrophosphate, *GGPP* geranylgeranyl pyrophosphate; Genes of enzymes are as follows: *ispA*, Farnesyl pyrophosphate synthase; *crtE*, GGPP synthase; *crtB*: phytoene synthase; *crtI*: phytoene desaturase; *crtY*: lycopene cyclase; PA*crtZ*, β-carotene hydroxylase from *P. agglomerans*; PC*crtZ*, *Paracoccus* sp. PC1; crtW*, mutated β-carotene ketolase; crtZ, β-carotene hydroxylase from *P. agglomerans* or *Paracoccus* sp. PC1

Astaxanthin, an important hydroxyl-carotenoid, has great potential commercial value in the feed, cosmetics, and nutraceutical industries due to its strong antioxidant capacity [6]. The heterologous synthesis pathway of astaxanthin comprises the two enzymes β-carotene ketolase (CrtW) [7] and β-carotene hydroxylase (CrtZ) [8, 9], which perform four interchangeable reactions starting from β-carotene and produce eight intermediates in addition to astaxanthin (Fig. 1). CrtZ is a bifunctional enzyme that catalyzes the hydroxylation reaction of β-carotene to yield zeaxanthin via β-cryptoxanthin, or the conversion of canthaxanthin into zeaxanthin via adonirubin [10]. These enzymes are crucial for the heterologous synthesis of hydroxyl-carotenoids, such as astaxanthin and zeaxanthin. To improve astaxanthin production, 12 β-carotene ketolases and 4 β-carotene hydroxylases from 5 cyanobacterial species were screened for astaxanthin biosynthesis in *E. coli*. The best recombinant strain reported in the literature expressed *crtZ* from *Pantoea agglomerans* and *crtW* from *Nostoc* sp. PCC 7120, producing an astaxanthin titter of 1.99 mg/g DCW [8]. Subsequently, 12 β-carotene hydroxylases genes, derived from archaea, bacteria, cyanobacteria, and plants, were investigated for their function about astaxanthin synthesis within *E. coli*. The result showed that *crtZ* from *P. agglomerans* was found to be the most suitable for astaxanthin biosynthesis. Moreover, efficient astaxanthin biosynthesis also requires the balanced expression of β-carotene ketolase and β-carotene hydroxylase [9]. Some studies showed that the adonixanthin, a monketolated derivative of zeaxanthin accumulated as the dominant intermediate [11, 12]. Zeaxanthin was found to be the only other detectable carotenoid in addition to astaxanthin, and reduced expression of *crtW* with respect to *crtZ* increased the efficiency of astaxanthin biosynthesis [13]. Conversely, canthaxanthin and echinenone were dominant products in addition except for astaxanthin, and their accumulation was reduced by increasing the gene copy number of *crtZ* [14]. We hypothesized that different ratios of intermediates were accumulated in the process of astaxanthin synthesis because enzymes from different sources have different substrate specificity [11–14]. The catalytic activities of 3 *crtZ* orthologs from different organisms were compared in the presence of both utilizable substrates in *vitro* [10]. The results indicated that the CrtZ enzymes from *Paracoccus* sp. PC1 (formerly known as *Alcaligenes* sp. PC-1) and *Agrobacterium aurantiacum* preferentially converted canthaxanthin to astaxanthin, while CrtZ from the *Pantoea ananatis* (formerly *Erwinia uredovora*) preferentially catalyzed the formation of zeaxanthin from β-carotene. The specific enzyme activity of CrtZ from *Paracoccus* sp. PC1 was 28 pmol/h/mg protein when converted canthaxanthin into astaxanthin, and was 3.4 pmol/h/mg/ protein when β-carotene as substrate. Furthermore, CrtZ from *Paracoccus* sp. PC1 showed that canthaxanthin was the favored substrate for conversion, 6-fold for canthaxanthin compared to β-carotene, when both substrates were added to the enzyme preparation in an equal ratio. However, it was still unclear whether the same substrate preference was still present in *vivo*, or if the canthaxanthin preference of CrtZ could reduce canthaxanthin accumulation and improve astaxanthin production when combined with another β-carotene hydroxylase with β-carotene propensity.

Ast007 is an astaxanthin producing strain in which *crtZ* and *crtW* (including 2 copies of PA*crtZ* and 1 copy of crtW*) (Table 1) were coordinately expressed. Although there are many intermediates such as β-carotene, canthaxanthin and echinenone accumulated in Ast007, the astaxanthin yield did not increase even more copy of *crtZ* or *crtW* was added [14]. A large amount of ketolated intermediates accumulation probably implied there were not suitable β-carotene hydroxylase catalyzes the hydroxylation of carotenoid β-ionone rings with keto group. In this study, the preference of PCCrtZ in the conversion of canthaxanthin into astaxanthin was tested, and used to improve astaxanthin production by co-utilizing with PACrtZ from *P. agglomerans* in *E. coli*.

**Table 1:** *Escherichia coli* strains used in this work

Strains	Relative characteristics	Copies of *crtYZW* genes						Sources
		*crtY*	PC *crtZ*		PA *crtZ*	crtW*		
CAR026	ATCC 8739, M1–37::*dxs*, M1–46::*idi*, *ldhA*::P-trc::*crtEIB::ldhA* ,M1–46::*SucAB*,M1–46::*sdh*, M1–46::*talB*, mRSL-4::ispG, mRSL-14::*ispH, pta*::P-trc::*crtY::pta* ,poxB::P-trc::*MVA::poxB*	1	0		0	0		[14]
CAR032	CAR026, *mgsA*::P-trc::*crtY*	2	0		0	0		This work
Strains for producing astaxanthin								
Ast004	CAR026, *mgsA*::P-trc::crtZW*	1	0		1		1	[14]
Ast005	CAR026, *mgsA*::P-trc::crtYZW*	2	0		1		1	[14]
Gro-46	Ast005, *pflB::crtZ::M1-RBSL-9,gno::M1-46*	2	0		2		1	[14]
Ast014	Can004, *manA*::P-trc::*crtZ*	2	0		1		2	This work
Ast015	Can004, *manA*::P-trc:: *crtZ-crtZ*	2	0		2		2	This work
Ast016	Can004, *manA*::P-trc:: PC*crtZ*	2	1		0		2	This work
Ast017	Can004, *manA*::P-trc:: PC*crtZ-*PC*crtZ*	2	2		0		2	This work
Ast018	Ast017, pflB::P-trc::PC*crtZ*	2	3		0		2	This work
Ast019	Ast017, pflB::P-trc::PC*crtZ-*PC*crtZ*	2	4		0		2	This work
Ast010	Gro-46,*manA*::P-trc:: PCcrtZ-crtW*	2	1		2		2	This work
Strains for producing zeaxanthin								
Zea001	CAR032, *manA*::P-trc::PC*crtZ*	2	1		0		0	This work
Zea002	CAR032, *manA*::P-trc::PC*crtZ-*PC*crtZ*	2	2		0		0	This work
Zea003	CAR032, *manA*::P-trc::*crtZ*	2	0		1		0	This work
Zea004	CAR032, *manA*::P-trc::*crtZ-crtZ*	2	0		2		0	This work
Strains for producing canthaxanthin								
Can003	CAR026, *mgsA*::P-trc::crtYW*	2		0	0		1	This work
Can004	Can003,*lacZ*::P-trc::crtW*	2		0	0		2	This work

## Results

### Construction of platform strains to confirm the functional expression of ***crtZ***

Beta-carotene hydroxylase (CrtZ) introduces hydroxyl moieties at the 3, 3’ position of the β-ionone ring irrespective of the presence or absence of a keto group at the 4, 4’ position. In order to study the substrate preference of different CrtZ orthologs, platform strains that produce high levels of β-carotene (substrate without keto group) or canthaxanthin (substrate with keto group) were constructed.

**Fig. 2:**
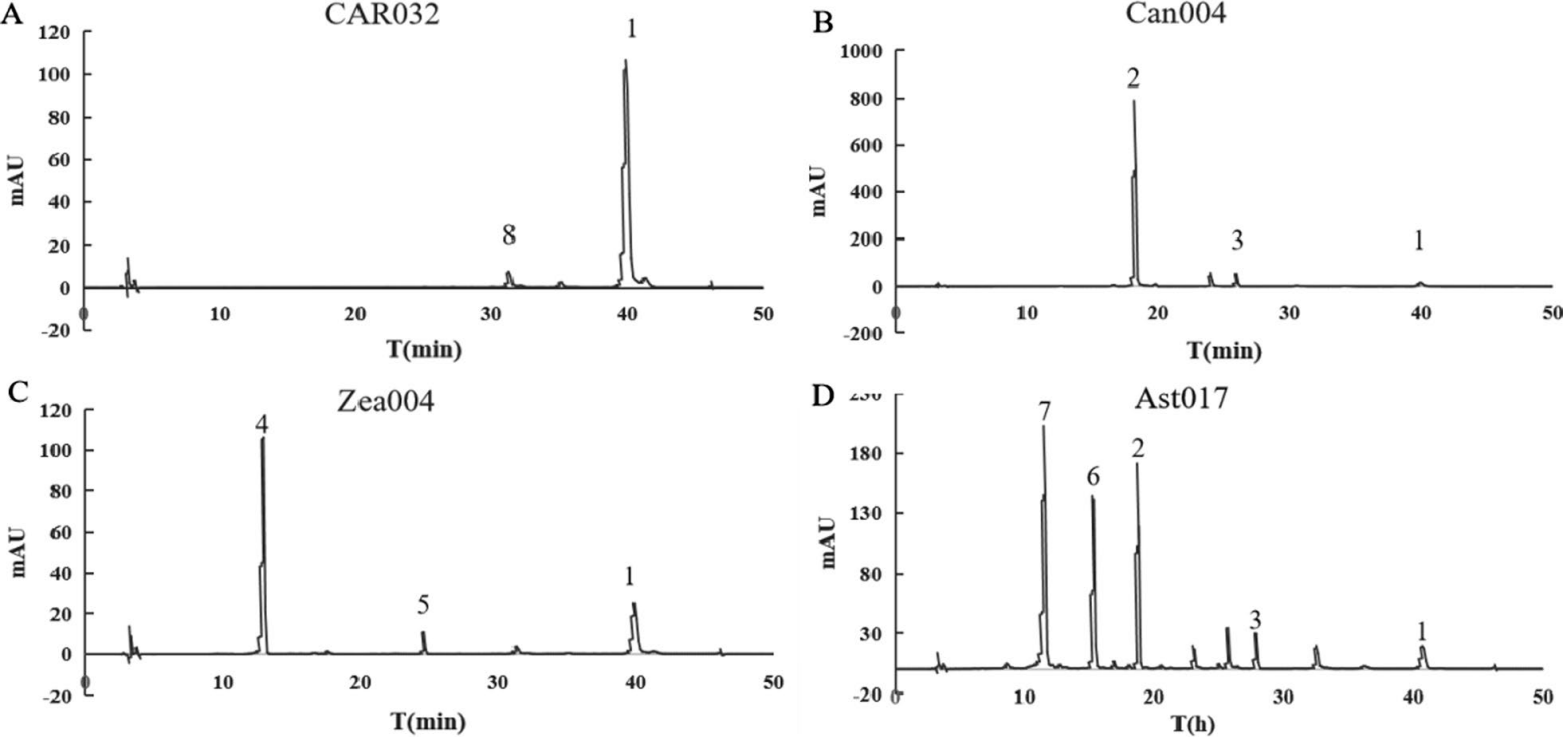
The peaks of carotenoids in the HPLC chromatogram

The numbers indicate the peaks of carotenoids in the HPLC chromatogram: 1, β-carotene; 2, canthaxanthin; 3, echinenone; 4, zeaxanthin; 5, β-cryptoxanthin; 6, adonixanthin; 7, astaxanthin; 8, lycopene.

The pathway fluxes of CAR026 were fully balanced by modular pathway optimization and β-carotene production of CAR026 reached to 3.6 g/L after 48 h of fed-batch of fermentation, with a little lycopene accumulation [14–17]. Ast004, in which one copy of PA*crtZ* and a copy of crtW* were integrated into the chromosome of CAR026 at *mgsA* site, had much more lycopene accumulation then that of CAR026, even there wasn’t any modification on lycopene biosynthesis pathway [14]. A large amount of lycopene accumulation in Ast004 was due to the insufficient activity of CrtY. Accordingly, additional copies of *crtY* can greatly reduce the accumulation of lycopene and increase the yield of astaxanthin [14]. Therefore, the β-carotene producing stain CAR032 was constructed by inserting a copy of *crtY* gene into the chromosome of CAR026 at the *mgsA* site. The biomass and β-carotene production of CAR032 respectively decreased by 16 and 4% compared to that of CAR026 in shake flasks fermentation (Table 2; Fig. 2 A). We speculated that the β-carotene accumulation inhibited lycopene synthesis [14]. Therefore, the addition of *crtY* did not improve the production of β-carotene due to the lack of lycopene. At the same time, biomass accumulation decreased because of an increased metabolic burden. However, additional copies of *crtY* could compensate for the insufficient activity of CrtY when the feedback was released because the β-carotene was converted into adonirubin or zeaxanthin [14]. Consequently, CAR032 was used as a platform to study the enzymatic properties of CrtZ orthologs from different organisms.

**Table 2:** Carotenoids production by representative *E. coli* strains

Strain^a^	OD_600_	canthaxanthin (mg/L)	echinenone (mg/L)	β-carotene (mg/L)
CAR026	6.96 ± 0.13	n.d.	n.d.	48.4 ± 3.47
CAR032	5.13 ± 0.08	n.d.	n.d.	46.51 ± 1.85
Can003	9.98 ± 0.72	26.6 ± 2.59	4.21 ± 1.73	8.62 ± 1.74
Can004	9.82 ± 0.26	40.7 ± 0.41	3.6 ± 0.65	3.87 ± 0.65

^a^Three repeats were performed for each strain, and the error bars represented standard deviation

CrtW converts β-carotene into keto-carotenoids, which have a keto group at the 4, 4 ' position of the ionone ring, and it can be used to synthesize canthaxanthin, which in turn can be converted into astaxanthin by CrtZ. The canthaxanthin producing strain Can003 was constructed by inserting a crtYW* operone at the *mgsA* site of CAR026 (Table 1). Can003 produced 26.6 mg/L of canthaxanthin, with 4.21 mg/L of echinenone and 8.62 mg/L of β-carotene as by-products (Table 2). This result demonstrated that more CrtW was needed to efficiently convert echinenone and β-carotene into canthaxanthin. An additional copy of crtW* was inserted into the chromosome at the *lacZ* site. The resulting strain Can004 produced 40.7 mg/L of canthaxanthin, which was 53% higher than the yield of Can003 (Tables 1, 2, Fig. 2A). At the same time, there was a significant reduction in the accumulation of the byproducts echinenone (from 4.21 to 3.6 mg/L) and β-carotene (from 8.62 to 3.87 mg/L). Consequently, Can004 was used as a platform to study the enzymatic properties of CrtZ from different organisms.

### **The substrate preference of PA*crtZ*and PC*crtZ***

To investigate the substrate preference of PA*crtZ* and PC*crtZ*, the corresponding genes were integrated into the *manA* sites of CAR032 and Can004, respectively. Two copies of PA*crtZ* were used in Ast007 to increase astaxanthin production and decrease by-product accumulation as described before [14]. Therefore, one and two copies of PA*crtZ* and PC*crtZ* were inserted into the *manA* sites of CAR032 and Can003, resulting in the strains Zea001-004 and Ast014-017, respectively (Additional file 1: Fig S1, Table 1).

**Fig. 3:**
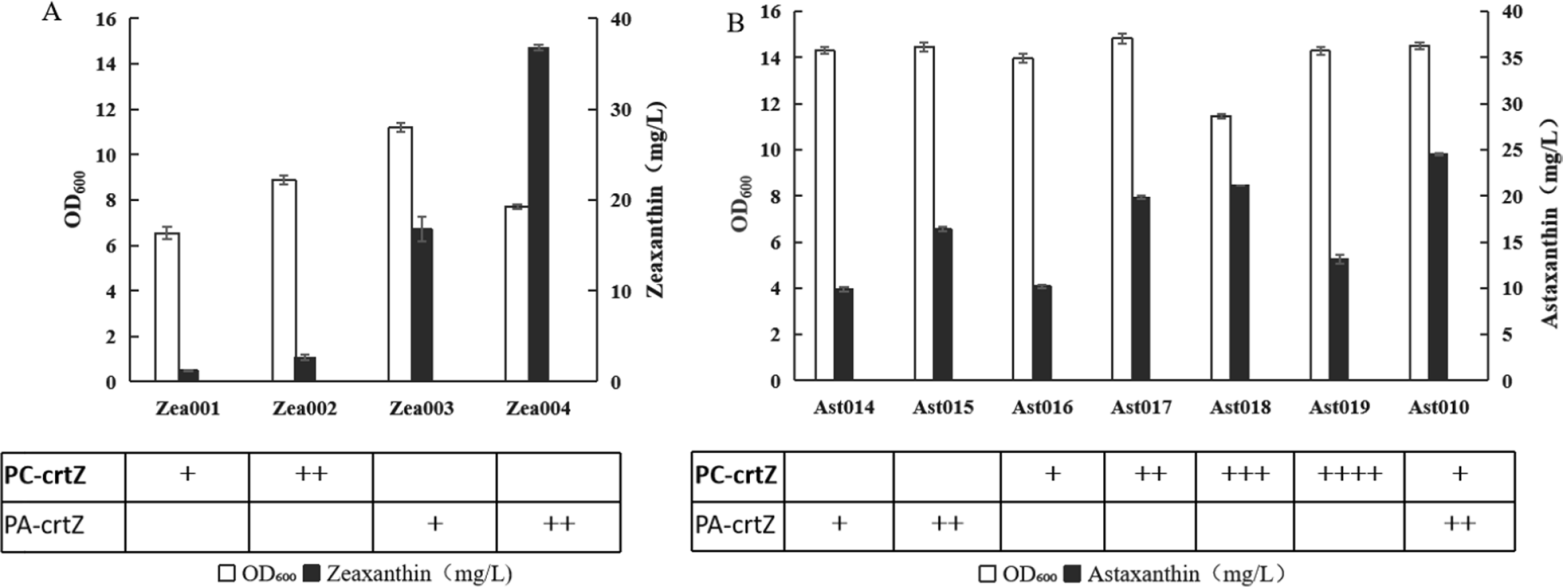
Expression PC*crtZ* and PA*crtZ* in platform strains. Three independent shake-flask fermentations were performed for each strain, and the error bars represent the standard deviations. A Cell growth and zeaxanthin production B Cell growth and astaxanthin production. The numbers of “+” in the tables indicate the numbers of corresponding *crtZ* in strains

Zea001 (CAR032 with one copy of PC*crtZ*) and Zea002 (CAR032 with two copies of PC*crtZ*) respectively produced 1.23 and 2.7 mg/L of zeaxanthin, while leaving a large amount of β-carotene (Fig. 3A, Additional file 1: Table S3). After integrating of one or two copies PA*crtZ*, the resulting strains produced 16.78 and 36.74 mg/L zeaxanthin, respectively accounting for 21 and 93% of the total carotenoids (Figs. 2C, 3A, Additional file 1: Table S3). PA*crtZ* from *P. agglomerans* therefore exhibited a substrate preference for β-carotene. On the contrary, PC*crtZ* from *Paracoccus* sp. PC1 showed weak activity in the conversion of β-carotene into zeaxanthin.

Ast014 (Can004 with one copy of PA*crtZ*) and Ast016 (Can004 with one copy of PC*crtZ*) produced similar amounts of astaxanthin, which were 9.9 and 10.21 mg/L astaxanthin respectively (Fig. 3B, Additional file 1: Fig. S2). Ast017 (Can004 with two copies of PC*crtZ*) and Ast015 (Can004 with two copies of PA*crtZ*) produced 19.84 mg/L and 16.4 mg/L astaxanthin, respectively. The astaxanthin production of Ast017 was 21% higher than that of Ast015 (Fig. 3B, Additional file 1: Fig. S2).

**Fig. 4:**
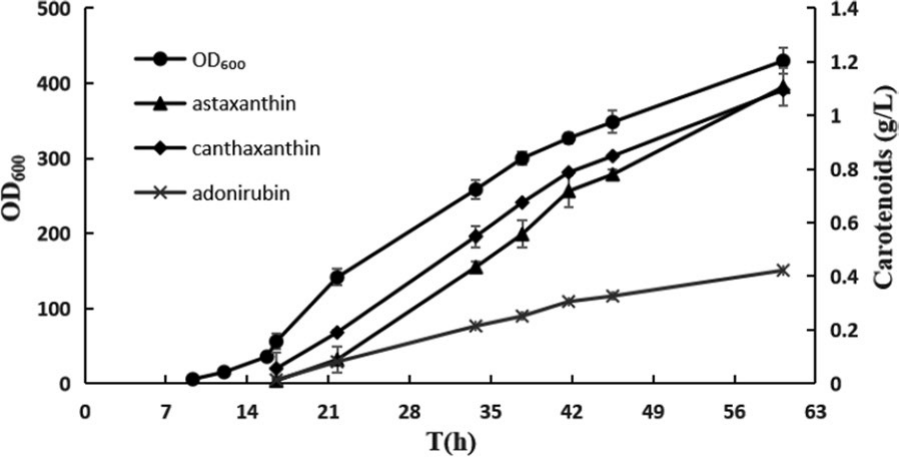
Fed-batch fermentation results for astaxanthin production by Ast0017. Three independent experiments were performed for each strain, and the error bars represent the standard deviations

The different culture conditions in shake flasks and a 5-L fermenter may lead to differences of product accumulation. To test this, Ast017 was cultivated in a 5-L fermenter at pH 7.0 to increase astaxanthin production and assess the by-product accumulation of adonirubin, canthaxanthin, echinenone or β-carotene. Strain Ast017 produced 1.1 g/L and 8 mg/g DCW of astaxanthin within 60 h (Fig. 4), in addition to 1.1 g/L of canthaxanthin and 0.42 g/L of adonirubin as predominant by-products (Fig. 2D). Canthaxanthin and adonirubin are both substrates of CrtZ and could be converted into astaxanthin by this enzyme, which demonstrated that the expression of PC*crtZ* in Ast017 was insufficient.

### **Improving astaxanthin production by integrating additional copies of PC*crtZ***

The results of shake-flasks and bioreactor fermentation showed that two copies of PC*crtZ* were insufficient to convert all intermediates into astaxanthin. Accordingly, another copy or two copies of PC*crtZ* were integrated into the chromosome of Ast017 to obtain Ast018 and Ast019, respectively (Additional file 1: Fig. S1, Table 1). Ast018 produced 21.1 mg/L of astaxanthin, which was 21% higher than the yield of Ast017 (Fig. 3B, Additional file 1: Fig. S2). However, Ast019 (Ast017 with the addition of two copies of PC*crtZ*) produced 13.12 mg/L of astaxanthin, which was a 34% decrease compared with Ast017 (Fig. 3B, Additional file 1: Fig. S4). The fourth copy of PC*crtZ* presumably caused an increasing metabolic burden [18], which led to reduce the yield of astaxanthin under shake flasks conditions.

Gro046 was the best astaxanthin producing strain we constructed before and produced 1.18 g/L astaxanthin after 60 h of fed-batch fermentation, in which *crtZ* and *crtW* (including 2 copies of *PAcrtZ* and 1 copy of crtW*) (Table 1) were coordinately expressed and the molecular chaperone genes *groES-groEL* were regulated [14]. To investigate the effect of combined utilization of PC*crtZ* and PA*crtZ* on the astaxanthin yield, a copy of PC*crtZ* was inserted into the chromosome of Gro-46(Fig.S1, Table 1) [14]. Furthermore, a copy of crtW* was inserted into the chromosome of Gro-46 together with PC*crtZ* in case crtW* insufficiency affects the astaxanthin yield, since Ast017 contains two copy of crtW* while Gro-46 only has a copy of crtW*. The resulting strain Ast010 produced 24.5 mg/L of astaxanthin which was 16% higher than the yield of Ast018 (Fig. 3B, Additional file 1: Fig. S2).

## Fed-batch fermentation of strains Ast018 and Ast010 for astaxanthin productionFed-batch fermentation of strains Ast018 and Ast010 for astaxanthin production

**Fig. 5:**
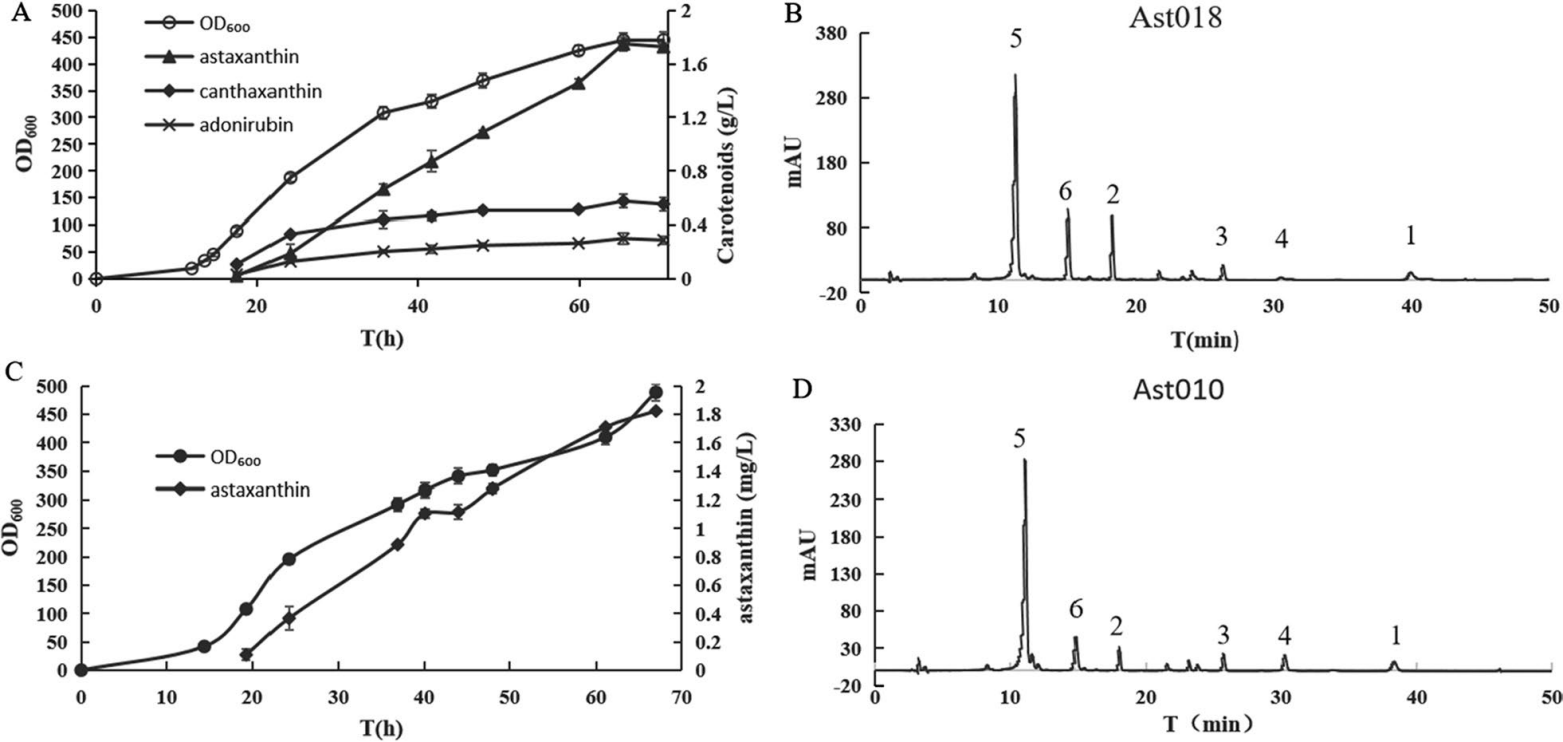
Fed-batch fermentation results for astaxanthin production by strains Ast018 and Ast010. Three independent experiments were performed for each strain, and the error bars represent the standard deviations. A Strain Ast018; B HPLC chromatograms of carotenoids produced by the strains Ast018; C Strain Ast010; D HPLC chromatograms of carotenoids produced by the strain Ast010. 1, β-carotene; 2, canthaxanthin; 3, echinenone; 4, lycopene; 5, astaxanthin; 6, adonixanthin

To further increase astaxanthin production, fed-batch fermentation of Ast018 and Ast010 was performed in a 5-L fermenter at pH 7.0. Ast018 produced 1.73 g/L and 12 mg/g DCW of astaxanthin within 70 h (Fig. 5AB), representing 57% and 50% increases over Ast0017, respectively. Additionally, 0.55 g/L of canthaxanthin and 0.29 g/L of adonirubin were accumulated as the major by-products, which was respectively 50% and 31% less than those in Ast017 (Fig. 5AB).

After 60 h of fed-batch fermentation, strain Ast010 produced 1.71 g/L and 12.9 mg/g DCW of astaxanthin (Fig. 5C), which was 45% and 65% higher than in Gro46 [14], respectively. The highest production of astaxanthin was 1.82 g/L after 67 h of fed-batch fermentation. Astaxanthin accounted for 65% of the total carotenoids and only small peaks representing other carotenoids appeared in the HPLC chromatogram (Fig. 5D).

## Discussion

The most suitable biosynthetic genes that enable the efficient channeling of available metabolic intermediates towards the desired product are important for the *in vivo* biosynthesis of complex natural products in a heterologous host. The catalytic activity of astaxanthin-producing enzymes derived from various species was compared in numerous studies [8, 9, 19–21]. Fraser et al. investigated characterization of the marine bacteria CrtZ and CrtW enzymes specific to astaxanthin biosynthesis and compared with the CrtZ from *P. anantis* and CrtW from *Haematococcus* BKT *in vitro* respectively. The results indicated that the CrtZ enzymes from *Paracoccus* sp. PC1 and *A. aurantiacum* preferentially converted canthaxanthin to astaxanthin, whereas the CrtZ from *P. anantis* had a propensity for the formation of zeaxanthin from β-carotene [10]. Furthermore, substrate inhibition of *crtZ* was observed at high substrate concentrations [22]. In this study, Zea004 which has two copies of PA*crtZ* produced high level of zeaxanthin (36.74 mg/L in shake flasks). Notably, *P. agglomerans* and *P. anantis* belong to the same genus, so the PA*crtZ* has similar enzymatic characteristics to *crtZ* from *P. anantis* (β-carotene preference and substrate inhibition). Furthermore, high levels of canthaxanthin (40.7 mg/L in shake flask fermentation and 3.9 g/L in fed-batch fermentation) were produced by Can004, which has two copies of crtW*(Additional file 1: Fig. S3). The results demonstrated that *crtW* from *Brevundimonas sp.* SD212 has a preference for β-carotene. Astaxanthin accounted for 53% of the total carotenoids produced by Gro-46 (peak area of astaxanthin / peak area of total carotenoid), and the major by-products of adonirubin (8%), canthaxanthin (5%), echinenone (5%), lycopene (12%) and β-carotene (17%) were accumulated besides astaxanthin. The low-level accumulation of zeaxanthin and adonixanthin may indicate insufficient CrtZ expression in Gro-46 [14]. However, additional copies of PA*crtZ* did not increase the astaxanthin yield. We speculated that PA*crtZ* preferentially converts β-carotene into zeaxanthin, and cannot easily convert ketocarotenoids into astaxanthin. Accordingly, the low ratio of astaxanthin to total carotenoids in Gro-46 was probably caused by the substrate preference and substrate inhibition of PA*crtZ*. To assess its substrate preference, PC*crtZ* was expressed in platform stains producing β-carotene and canthaxanthin, respectively. Low levels of zeaxanthin was got by expressing PC*crtZ* in CAR032, while high levels of astaxanthin was produced by expressing PC*crtZ* in Can004, which suggested that PC*crtZ* has a substrate preference for canthaxanthin over β-carotene, which was consistent with earlier reports.

Ast018 with three copies of PC*crtZ* and two copies of *crtW* produced 1.73 g/L of astaxanthin, while Ast019 containing four copies of PC*crtZ* and two copies of crtW* produced 1.75 g/L of astaxanthin along with certain amounts of canthaxanthin and adonirubin in fad-batch fermentation (Fig. 4, Additional file 1: Fig. S3). The fourth PC*crtZ* in Ast019 therefore did not reduce accumulation of canthaxanthin and adonirubin, presumably due to substrate inhibition.

To investigate the effect of combined utilization of PC*crtZ* and PA*crtZ* on the astaxanthin yield, PC*crtZ* and PA*crtZ* were co-expressed in Ast010. Ast010 produced 1.82 g/L and 11.5 mg/g DCW of astaxanthin after 67 h of fed-batch fermentation, whereby astaxanthin accounted for more than 65% of the total carotenoids. The peaks of all other carotenoids were much smaller than that of astaxanthin, demonstrating that the combination of CrtZ orthologs with complementary substrate preferences can effectively improve the yield and purity of astaxanthin. To our best knowledge this strategy was used for the synthesis of astaxanthin for the first time in this study.

It was reported that the coordinated expression of *crtZ* and *crtW* led astaxanthin as the only carotenoid product [13]. However, the peak area of astaxanthin among the carotenoid products of Ast010 was only 65% of the total. The low ratio of astaxanthin synthesized by Ast010 has two possible explanations. First, it is possible that some unusual intermediates were accumulated. Because the carotenoid synthesis pathway and central metabolic pathways of the platform strains used in this study were optimized [15, 16, 23], a large amount of lycopene and β-carotene were synthesized. Notably, small amount of γ-carotene was accumulated as a by-product of β-carotene synthesis (a small peak between 1 and 3 in Fig. 2A, the γ-carotene is undetectable when β-carotene production is low). The corresponding hydroxylated and ketylation products cannot be further converted to astaxanthin and consequently accumulated. Secondly, substrate inhibition makes the catalytic reaction incomplete. Substrate inhibition of CrtZ and CrtW was reported previously, blocking the further conversion of intermediate derived from β-carotene into astaxanthin. Higher substrate concentrations would lead to the accumulation of more intermediates such as adonirubin, canthaxanthin, and echinenone. Certainly, more efforts in modifying recombinant strain by metabolic engineering and optimizing fermentation condition may increase the yield and purity of astaxanthin.

Multiple copies of *crtZ* and crtW* were used to improve astaxanthin production in Ast010 and Ast017, which probably implied that the specific activity of CrtZ and CrtW* is low. Excessive overexpression of heterologous genes can lead to a metabolic burden or protein misfolding, as well as non specific side reactions [24]. Therefore, increasing the specific activity of CrtZ and CrtW*, and accordingly decreasing the copy number of *crtZ* and crtW* may be an effective strategy to increase astaxanthin production and reduce the accumulation of by-products. The PCCrtZ and PACrtZ enzymes convert the same substrates, but have different substrate preferences. In future studies, homology modeling and analysis of the active sites of these two enzymes, which may elucidate the structural basis of their substrate, could be used for further rational engineering of CrtZ by site-directed mutagenesis.

## Conclusions

To the best of our knowledge, we report the highest astaxanthin obtained in engineered *E. coli* to date. The combined utilization of CrtZ orthologs with different substrate preferences can enhance the production of astaxanthin and increase the ratio of astaxanthin among total carotenoids.

## Materials and methods

### Strains, media and growth conditions

All strains used in this study are listed in Table 1; their construction is depicted in Additional file 1: Fig. S1 and described in detail below. The plasmids used are presented in Additional file 1: Table S2, and primers in Additional file 1: Table S1. During strain construction, the cultures were grown aerobically at 30 °C, 37 °C, or 39 °C in Luria Bertani (LB) medium. For carotenoid production, single colonies were picked from the plate and grown in tubes containing 4 mL of LB at 30 °C and 250 rpm overnight. The resulting seed cultures were used to inoculate 100-mL flasks containing 20 mL of LB with 2% glycerol to an initial OD_600_ of 0.05, and grown at 30 °C and 250 rpm. After 24 h of growth, the cells were collected for measurement of carotenoid production.

### Construction of plasmids for the expression of heterologous ***crtZ*** genes

Plasmids were assembled using the Seamless Cloning Kit (Beyotime, China). To determine the effects of *crtZ* from different organisms on the production of astaxanthin, plasmids psc104-crtZ and psc104-PCcrtZ were constructed. The PA*crtZ* gene was amplified from *P. agglomerans* CGMCC No.1.2244 (GenBank accession number HQ003247) using the primer pair CrtZ-RBS-99 A-CPEC-F/CrtZ-CPEC-R (Additional file 1: Table S1), and the plasmid backbone was amplified from pSC103 using the primer pair 99 A-CPEC-F/99A-CPEC-R. Then, the two PCR fragments were ligated using the Seamless cloning kit (Beyotime), resulting in the plasmid pSC104-crtZ. The PC*crtZ* was synthesized by Genscript Inc according to the sequence of *crtZ* from *Paracoccus* sp. PC1 (GenBank accession number: D58422), and was amplified using the primer pair PC-crtZ-CPEC-RBS-F/PC-crtZ-CPEC-R (Additional file 1: Table S1). Then it was ligated with plasmid backbone, resulting in plasmid pSC104-PCcrtZ.

### Integration of the heterologous genes into the *E. coli* chromosome

All *E. coli* gene insertions were performed using two-plasmid CRISPR/Cas9 technology as reported previously [14, 25]. The construction of donor plasmids (Additional file 1: Table S2) were described in Additional file 1: Text S1.The primers used for gene integration is listed in Additional file 1: Table S1.

Integration of *crtZ* was described as an example to show how to integrate heterologous genes into the *E. coli* chromosome. The helper plasmid pCas9 and donor plasmid pManA-crtZ were co-electroporated into the β-carotene producing strain CAR032, and the resulting strain was processed using the Cas9 genome-editing protocol as described previously [25–27], yielding the strain Zea001 with a PC*crtZ* integration at the *manA* site. Zea002, Zea003, and Zea004 were obtained by inserting PC*crtZ-*PC*crtZ*, PA*crtZ* or PA*crtZ-*PA*crtZ* into the chromosome of CAR032 at the *manA* site respectively, using the same method.

### Fed-batch fermentation of strains Ast017, Ast018 and Ast010

Strains Ast017, Ast018 and Ast010 were used for producing astaxanthin through fed-batch fermentation. Two stage systems were used for preparing seed. The first seed was prepared by inoculating fresh colonies into a 100 mL flask containing 10 mL medium at 30℃ with shaking at 250 rpm until OD600 was 0.7-1.0. Then 3 mL of culture from first seed stage was added to a 500 mL flask containing 120 mL medium and incubated at 30℃ with shaking at 250 rpm until OD600 reached 1.5. The second seed solution was transferred to a 5 L fermentation tank containing 3 L substrate medium. The fermentation substrate medium and fermentation progress were the same as those described previously [14].

### Measurement of carotenoid production

Carotenoids were extracted using acetone as described before [15]. A sample comprising 200 µL of the culture broth was harvested by centrifugation at 13,800 × g for 5 min, resuspended in 1 mL of acetone, and incubated at 55 °C for 15 min in the dark. The samples were then centrifuged at 13,800 × g for 10 min to obtain the supernatant containing carotenoids, which was used for HPLC analysis.

The analytes were separated on a Symmetry C18 column (250 mm × 4.6 mm, 5 μm, Waters, Ireland), which was kept at 30 °C, and were detected at 476 nm using a Shimadzu UV-2550 spectrophotometer (Shimadzu, Kyoto, Japan). The mobile phase and flow rate in measurement of carotenoids production were the same as that described previously [14]. The results represent the means ± SD of three independent experiments. Dry cell weight (DCW) was calculated from the optical density at 600 nm (OD_600_) using the empirical formula 1 OD_600_ = 0.323 g DCW L^− 1^.

## Supplementary Information

**Additional file : Text S1** Construction of plasmids for the chromosomal integration of heterologous genes. **Text S2** The amino acid sequences of PCcrtZ. **Text S3** The amino acid sequences of PacrtZ. **Table S1.** Primers used in this study. **Table S2.** Plasmids used in this study. **Table S3.** Caretenoids production by strains producing Zeaxanthin in shake flasks. **Fig. S1.** Flowchart illustrating the construction of the main strains used in this study **Fig. S2.** Carotenoids production of antxanthin producing strains. **Fig. S3.** Fed-batch fermentation results for canthaxanthin production by strains Can004 **Fig. S4.** Fed-batch fermentation results for astaxanthin production by strains Ast019.

## Data Availability

All data generated or analysed during this study are included in this published article and its Additional fle 1: information fles.
