# Supplementary material for: Improving astaxanthin production in Escherichia coli by co-utilizing CrtZ enzymes with different substrate preference
Source: Microb Cell Fact. 2022 Apr 25;21:71. doi: 10.1186/s12934-022-01798-1 (PMC9036794; doi:10.1186/s12934-022-01798-1)
Supplement: Supplementary file 1 — Additional file : Text S1 Construction of plasmids for the chromosomal integration of heterologous genes. Text S2 The amino acid sequences of PCcrtZ. Text S3 The amino acid sequences of PacrtZ. Table S1. Primers used in this study. Table S2. Plasmids used in this study. Table S3. Caretenoids production by strains producing Zeaxanthin in shake flasks. Fig. S1. Flowchart illustrating the construction of the main strains used in this study Fig. S2. Carotenoids production of antxanthin producing strains. Fig. S3. Fed-batch fermentation results for canthaxanthin production by strains Can004 Fig. S4. Fed-batch fermentation results for astaxanthin production by strains Ast019. [file 12934_2022_1798_MOESM1_ESM.docx]

Microbial Cell Factories 2022.3

**Supplementary information**

**Improving astaxanthin production in *Escherichia coli***

**by co-utilizing CrtZ enzymes with different substrate preference**

Meng Zhang^1,2 #^. Zhongkuo Gong^2,#^ . Jinlei Tang^2,3,4,5^. Fuping Lu^1^

QingYan Li^2,3,4,5*^.XueLi Zhang^2,3,4,5*^

**The full names, institutional addresses and email addresses for Correspondence authors:**

^1^ College of Biotechnology, Tianjin University of Sciences and Technology, Tianjin, 300457, China

^2^ Tianjin Institute of Industrial Biotechnology, Chinese of Academy of Sciences, Tianjin, 300308, China

^3^ Key Laboratory of Systems Microbial Biotechnology, Chinese Academy of Sciences, Tianjin, 300308, China

^4^ National Technology Innovation Center of Synthetic Biology, Tianjin, 300308, China

^5^ University of Chinese Academy of Sciences, Beijing, 100071, China

^*^Correspondence to. 32 Xiqidao, Tianjin Airport Economic Park, Tianjin, 300308, China.

^#^These authors contributed equally to this work.

**Text S1**

**Construction of plasmids for the chromosomal integration of heterologous genes**

Two-plasmid based CRISPR/Cas9 system (donor plasmid and helper plasmid) was used for integrating heterogous genes in the chromosome of recombinant *E. coli* in this study. The plasmid pManA-N20 and pPflB-N20 were constructed using the same method as pMgsA-N20 that was described before [1], which were used as backbone to construct plasmids used to integrate heterologous genes at the *manA* and *pflB* site respectively. The N20 sequence (AATGTGGTGAAATTGAATCC) was inserted into pManA-N20 on the primer manA-N20-F2, and the N20 sequence (ACCGAATCTATCGGTGGTAT) was inserted into pPflB-N20 on the primer pflB-N20-F2.

Construction of plasmids pManA-crtZ and pManA-PCcrtZ were described as an example to show how to obtain donor plasmids for genes integration. The plasmid pManA-N20 was used as the template for inside-out amplification using the primer pair ManA-F2 / ManA-R2 to get the plasmid backbone. The sequences including gene and trc promoter, trc-crtZ and trc-PCcrtZ, were amplified from pSC104-crtZ and pSC104-PCcrtZ using the primer pair Trc-manA-F /rrnB-manA-R. The trc-crtZ and trc-PCcrtZ were fused with plasmid backbone respectively, to obtain plasmids pManA-crtZ and pManA-PCcrtZ, which were used to integrate PA*crtZ* or PC*crtZ* together with trc promoter into the chromosome of CAR032 at the *manA* site. All plasmids for integration at *pflB*, *lacZ* sites were constructed using same method as pManA-crtZ, and are listed in Table S2. Primers used in this study are listed in Table S1.

To integrate two *crtZ* genes in one time, pManA-crtZ-crtZ was constructed. The plasmid pManA-crtZ was used as the template for inside-out amplification using the primer pair ManA-F2 /crtZ-taa-R to get the plasmid backbone. The PA*crtZ* was amplified using the primer pair crtZ-RBS-crtZ-F /rrnB-manA-R, and fused with plasmid backbone, resulting in plasmid pManA-crtZcrtZ. pManA-crtZ-crtZ included two *crtZ* ligated using RBS (AAGGAGATATACC) and controlled by trc promoter. The pManA-PCcrtZ-PCcrtZ was prepared in the similar way as pManA-crtZ-crtZ.

pMgsA-crtY and pMgsA-crtYW* were derived from pMgsA-crtYZW* by using CPEC method in the similar way as pManA-crtZ-crtZ.

Text S2 The amino acid sequences of PCcrtZ

MTQFLIVVATVLVMELTAYSVHRWIMHGPLGWGWHKSHHEEHDHALEKNDLYGVVFAVLA

TILFTVGAYWWPVLWWIALGMTVYGLIYFILHDGLVHQRWPFRYIPRRGYFRRLYQAHRL

HHAVEGRDHCVSFGFIYAPPVDKLKQDLKRSGVLRPQDERPS

Text S3 The amino acid sequences of PAcrtZ

MLWIWNALIVLVTVIGMEITAALAHRYIMHGWGWGWHLSHHEPHKGWFEVNDLYAVVFAA

LSILLIYLGSTGVWPLQWIGAGMTLYGLLYFIVHDGLVHQRWPFRYVPRRGYLRRLYMAH

RMHHAVRGKEGCVSFGFLYAPPLSKLQATLRERHGVKRGAARDQRSVERDAPPGK

Table S1 Primers used in this study.

| **Primers** | **Sequences** |
| --- | --- |
| **For plasmids pSC104-PCcrtZ and pSC104-crtZ** **construction** | |
| 99A-CPEC-F | TGTTTTGGCGGATGAGAGAA |
| 99A-CPEC-R | GGTCTGTTTCCTGTGTGAAATTG |
| CrtZ-RBS-99A-CPEC-F | CAATTTCACACAGGAAACAGACCAAGGAGATATACC ATGTTGTGGATTTGGAATGCCCT |
| CrtZ-CPEC-R | TTCTCTCATCCGCCAAAACATTACTTCCCGGGTGGCGC |
| PC-crtZ-CPEC-R | TTCTCTCATCCGCCAAAACATCACGACGGACGCTCGTC |
| PC-crtZ-CPEC-RBS-F | CAATTTCACACAGGAAACAGACCAAGGAGATATACC ATGACGCAATTCCTCATTGTCG |
| **For plasmids construction used for gene integration at *manA* site** | |
| N20-B-F1: | CCAGGTCTCAGTGCGCCATGAGAACGAACCATTG |
| N20-B-R1: | CCAGGTCTCAGCTAAGATCTGACTCCATAACAGAGTACTCGC |
| manA-N20-F2 | CCAGGTCTCATAGCAATGTGGTGAAATTGAATCCGTTTTAGAGCTAGAAATAGCAAGTTAAAATAAGGC |
| N20-B-R2: | CCAGGTCTCAACCGCTGGCTAAATACGGAAGGATCT |
| ManA-bsaI-F1 | CCAGGTCTCACGGT CTTGTTTGCCGAACCACCAC |
| ManA-bsaI-R1 | CCAGGTCTCAGCAC AACTGCGTCTATCCGACCACT |
| ManA-R2 | GGCATTCTGCACTCGTGAAC |
| ManA-F2 | GCAGTTACAGCTTAAACCGGGT |
| Trc-manA-F | AAAGCAGTTCACGAGTGCAGAATAATGCTTCTGGCGTCAGGCA |
| rrnB-manA-R | TCACCCGGTTTAAGCTGTAACTGCAATCTTCTCTCATCCGCCAAAAC |
| crtZ-RBS-crtZ-F | GTGACGCGCCACCCGGGAAGTAATAAGGAGATATACCATGACCGCCGCAGTCACAGA |
| crtZ-taa-R | TTACTTCCCGGGTGGCGC |
| PCcrtZ-RBS-crtZ-F | GTGACGCGCCACCCGGGAAGTAATAAGGAGATATACCATGACGCAATTCCTCATTGTCG |
| PCcrtZ-taa-R | TCACGACGGACGCTCGTC |
| **For plasmids construction used for gene integration at *pflB* site** | |
| pflB-bsaI-CGGT-F1 | CCAGGTCTCACGGT CTGCGGAGCCGATCTCTTTAC |
| pflB-bsaI-GCAC-R1 | CCAGGTCTCAGCACCGAGTAATAACGTCCTGCTGCT |
| pflB-N20-F2 | CCAGGTCTCATAGCACCGAATCTATCGGTGGTATGTTTTAGAGCTAGAAATAGCAAGTTAAAATAAGGC |
| pflB-F2 | AAACGGGTAACACCCCAGAC |
| pflB-R2 | GCTCGGACATGTAACACCTACC |
| Trc-pflB-F | GAAGGTAGGTGTTACATGTCCGAGCAATGCTTCTGGCGTCAGGCA |
| rrnB-pflB-R | GACCGTCTGGGGTGTTACCCGTTTAATCTTCTCTCATCCGCCAAAAC |
| **For plasmids construction used for gene integration at *lacZ* site** | |
| lacZ-F2 | AATAACCGGGCAGGCCAT |
| lacZ-R2 | GTTTCCTGTGTGAAATTGT |
| Trc-lacZ-F | GATAACAATTTCACACAGGAAACAATGCTTCTGGCGTCAGGCA |
| rrnB-lacZ-R | CAGACATGGCCTGCCCGGTTATTAATCTTCTCTCATCCGCCAAAAC |
| **For plasmids construction used for gene integration at *mgsA* site** | |
| CrtY-R | TTATTGCATCGCCTGTTGACG |
| MgsA-crtY-F | CACCGTCAACAGGCGATGCAATAA GTTATCTCGCGGACCGTCTG |
| CrtW*-RBS-crtY-F | CACCGTCAACAGGCGATGCAATAA AAGGAGATATACC ATGTTGTGGATTTGGAATGCCCT |

Table S2 Plasmids used in this study.

| **Plasmids** | **Relative characteristics** | **Sources** |
| --- | --- | --- |
| pKD46 | bla*γ β exo* (Red recombinase), temperature-conditional replicon | [[2]](file:///E:\可移动磁盘J\萜类化合物研究\2016.12虾青素-李\虾青素发文章计划\虾青素文章crtZ筛选\文章文档\manucript-crtZ-插图.docx#_ENREF_6) |
| pSC103 | *cat,* Low copy plasmid, ori and rep A from p SC102, bla; trc Promoter with RBS AAGGAGATATACC and rrn terminator from pTrc99A, cat from p ACYC184 | Lab collection |
| pMgsA-crtYZW* | *cat*; trac promoter followed by *crtY, crtZ* and *crtW** amplified from pSC104-YZW* cloned into pMgsA-N20 | [[1]](file:///E:\可移动磁盘J\萜类化合物研究\2016.12虾青素-李\虾青素发文章计划\虾青素文章crtZ筛选\文章文档\manucript-crtZ-插图.docx#_ENREF_9) |
| pUC57-PCcrtZ | *Amp, crtZ* from *Paracoccus* sp. PC1 was synthesized by Genscript Inc and cloned into pUC57 | This work |
| pSC104-PCcrtZ | *cat, crtZ* amplified from pUC57-PCcrtZ cloned into pSC103 | This work |
| pSC104-crtZ | *cat, crtZ* amplified from *Pantoea agglomerans* cloned into pSC103 | This work |
| pSC104-crtY | *cat, crtY* from *P. agglomerans* cloned into pSC103 | This work |
| pSC104-crtY-crtW* | *cat, crtY* from *P. agglomerans* and *crtW** from Ast005 cloned into pSC103 | This work |
| pSC104-crtW* | *cat,* *crtW** from Ast005 cloned into pSC103 | This work |
| pCas9 | *Cas9* | [[3]](file:///E:\可移动磁盘J\萜类化合物研究\2016.12虾青素-李\虾青素发文章计划\虾青素文章crtZ筛选\文章文档\manucript-crtZ-插图.docx#_ENREF_10) |
| pRed_Cas9 | *Kan，*Derived from pKD46, exo, bet, gam, arabinose operon, Cas9 from , pCas9, temperature-conditional replicon | [[4]](file:///E:\可移动磁盘J\萜类化合物研究\2016.12虾青素-李\虾青素发文章计划\虾青素文章crtZ筛选\文章文档\manucript-crtZ-插图.docx#_ENREF_24) |
| pACYC184-gRNA | *cat,* Derived from pACYC184-M, gRNA with N20 | [[1]](file:///E:\可移动磁盘J\萜类化合物研究\2016.12虾青素-李\虾青素发文章计划\虾青素文章crtZ筛选\文章文档\manucript-crtZ-插图.docx#_ENREF_9) |
| pMgsA-N20 | *cat,* Derived from pACYC184-gRNA, gRNA with N20 and homologous arms of *mgsA* | [[1]](file:///E:\可移动磁盘J\萜类化合物研究\2016.12虾青素-李\虾青素发文章计划\虾青素文章crtZ筛选\文章文档\manucript-crtZ-插图.docx#_ENREF_9) |
| pManA-N20 | *cat,* Derived from pACYC184-gRNA, gRNA with N20 and homologous arms of *manA* | This work |
| placZ | *cat, P15A* from pACYC184-M, lacZ-N20-gRNA targeting *lacZ* gene expressed under constitute promoter, *lacZ* gene, its up and down homologous hand | [5] |
| pPflB-N20 | *cat,* Derived from pACYC184-gRNA, gRNA with N20 and homologous arms of *pflB* | This work |
| pMgsA-crtY | *cat*; trac promoter followed by *crtY* amplified from *P. agglomerans* cloned into pMgsA-N20 | This work |
| pMgsA-crtYW* | *cat*; trac promoter followed by *crtY* from *P. agglomerans* and *crtW** from Ast005 cloned into pMgsA-N20 | This work |
| pLacZ-PCcrtZ | *cat*; trac promoter followed by *crtY* from *P. agglomerans* and *crtW** from Ast005 cloned into pMgsA-N20 | This work |
| pLacZ-crtW* | *cat*; trac promoter followed by *crtW** from Ast005 cloned into pLacZ-N20 | This work |
| pManA-crtZ | *cat*; trac promoter followed by *crtZ* from *P. agglomerans* cloned into pManA-N20 | This work |
| pManA-crtZ-crtZ | *cat*; trac promoter followed by two *crtZ* from *P. agglomerans* linker by a RBS sequence cloned into pManA-N20 | This work |
| pManA-PCcrtZ | *cat*; trac promoter followed by *crtZ* from *Paracoccus* sp. PC1cloned into pManA-N20 | This work |
| pManA-PCcrtZ-PCcrtZ | *cat*; trac promoter followed by two *crtZ* from *Paracoccus* sp. PC1 linker by a RBS sequence cloned into pManA-N20 | This work |
| pPflB-PCcrtZ | *cat*; trac promoter followed by *crtZ* from *Paracoccus* sp. PC1cloned into pPflB-N20 | This work |
| pPflB-PCcrtZ-PCcrtZ | *cat*; trac promoter followed by two *crtZ* from *Paracoccus* sp. PC1 linker by a RBS sequence cloned into pPflB-N20 | This work |

Table S3 Caretenoids production by strains producing Zeaxanthin in shake flask.

| Strains^a^ | Zeaxanthin^b^ | β-Cryptoxanthin | β-carotene | Zeaxanthin/Carotenoids |
| --- | --- | --- | --- | --- |
| Zea001 | 63.7±4.37 | 68.4±4.61 | 2531±110.98 | 3% |
| Zea002 | 158.8±17.62 | 119.5±5.73 | 3917.2±184.83 | 4% |
| Zea003 | 1072.0±68.12 | 326.0± 20.1 | 4053.6±20.38 | 21% |
| Zea004 | 2369.1±14.38 | 64.6±7.5 | 112.0±11.24 | 93% |

^a^ Three repeats were performed for each strain, and the error bars represented standard deviation.

^b^ The peak area was calculated based on the dilution factor, which represents the mounts of carotenoids in 200 µl of cell broth pellet measured by HPLC.

Reference

1. Gong Z, Wang H, Tang J, Bi C, Li Q, Zhang X. Coordinated Expression of Astaxanthin Biosynthesis Genes for Improved Astaxanthin Production in *Escherichia coli*. J Agric Food Chem. 2020; 68:14917-14927.

2.Datsenko KA, Wanner BL. One-step inactivation of chromosomal genes in *Escherichia coli* K-12 using PCR products. Proc Natl Acad Sci USA. 2000; *97* (12): 6640-6645

3. Jiang, W.; Bikard, D.; Cox, D.; Zhang, F.; Marraffini, L. A., RNA-guided editing of bacterial genomes using CRISPR-Cas systems. Nat Biotechnol. 2013; *31* (3): 233-239.

4. Zhao, D.; Yuan, S.; Xiong, B.; Sun, H.; Ye, L.; Li, J.; Zhang, X.; Bi, C., Development of a fast and easy method for *Escherichia coli* genome editing with CRISPR/Cas9. Microb Cell Fact. 2016; *15* (1): 205.

5. Qiu H, Zhao D, Li S, Bi C, Zhu X, Zhang X. Construction of promoters with tight regulation on chromosome of *Escherichia coli*. Microbiol China. 2018; 45(8): 1693−1704.

Supplementary Figures


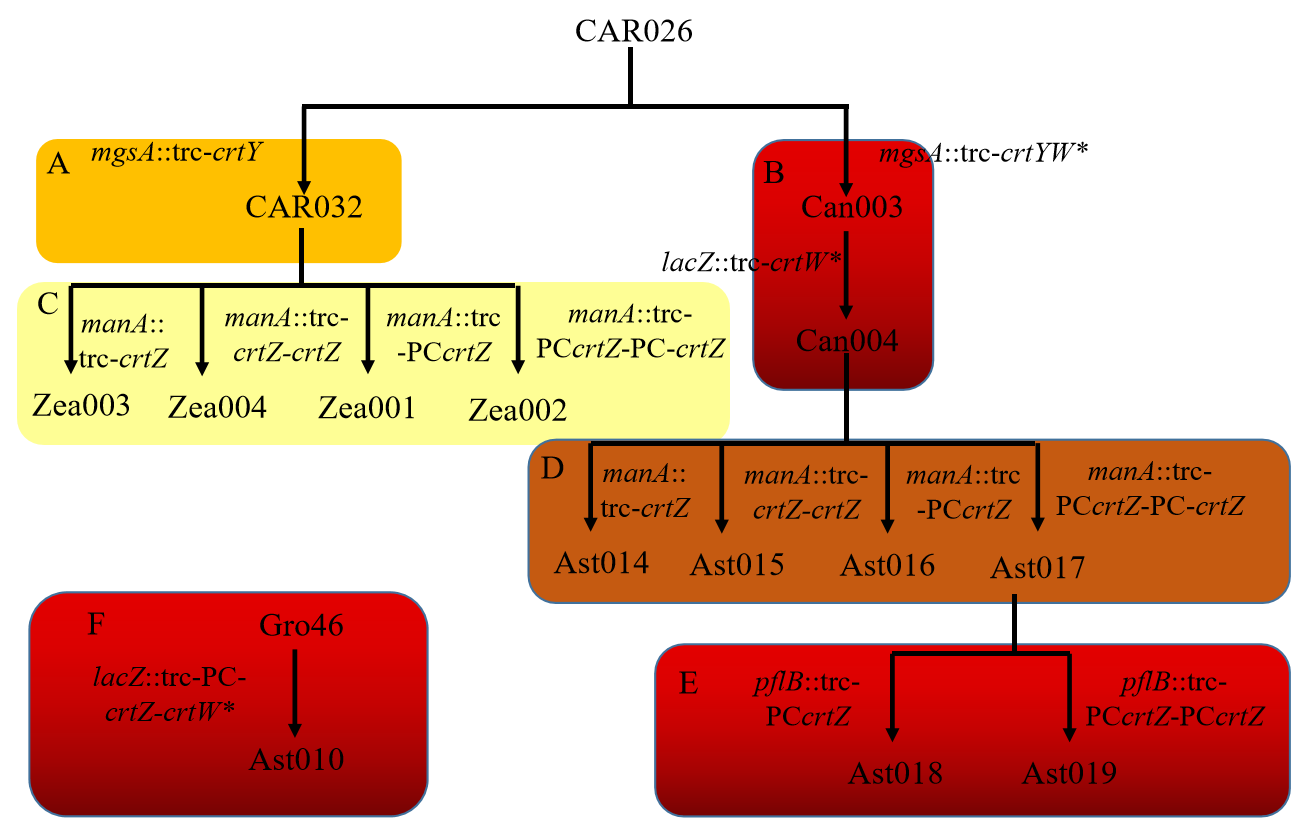


Fig. S1 Flowchart illustrating the construction of the main strains used in this study. A: Another copy of *crtY* was inserted in the chromosome of CAR026; B: construction of strains producing canthaxanthin; C: Chromosomal integration of one copy or two copies of *crtZ* at the *manA* locus of CAR032; D: Chromosomal integration of one copy or two copies of *crtZ* at the *manA* locus of Can004; E: Chromosomal integration of one copy or two copies of PC*crtZ* at the *pflB* locus of Ast017; F: Chromosomal integration of a copy of PC*crtZ* and a copy of *crtW** at the *lacZ* locus of Gro46.

Fig. S2 Carotenoids production of antxanthin producing strains. Three independent shake-flask fermentations were performed for each strain, and the error bars represent the standard deviations. Abbreviations: Ast：astaxanthin Ado: adonixanthin Ech: echinenone Can: canthaxanthin Car：β-carotene

Fig. S3 Fed-batch fermentation results for canthaxanthin production by strains Can004

Fig **S4.** Fed-batch fermentation results for astaxanthin production by strains Ast019
